# False-negative CMV PCR results due to viral sequence variation: a diagnostic pitfall with the potential for serious consequences

**DOI:** 10.1128/asmcr.00159-25

**Published:** 2025-11-17

**Authors:** Huanyu Wang, Monica I. Ardura, Sophonie J. Oyeniran, Amy L. Leber

**Affiliations:** 1 Department of Pathology and Laboratory Medicine, Nationwide Children’s Hospital, Columbus, Ohio, USA; 2 Department of Pathology, The Ohio State University 2647, Columbus, Ohio, USA; 3 Department of Pediatrics, Division of Infectious Diseases, Host Defense Program, Nationwide Children’s Hospital and The Ohio State University 2647, Columbus, Ohio, USA; 4 Department of Pediatrics, Division of Infectious Diseases, Nationwide Children’s Hospital and The Ohio State University 2647, Columbus, Ohio, USA; Rush University Medical Center, Chicago, Illinois, USA

**Keywords:** transplant, CMV, NAAT, immunocompromised, false negative

## Abstract

**Background:**

Cytomegalovirus (CMV) continues to be a significant cause of morbidity and mortality in immunocompromised patients. Nucleic acid amplification tests (NAATs) are the preferred method for both diagnosing CMV infection and monitoring response to antiviral therapy in these patients. The use of a sensitive and specific CMV NAAT is essential to ensure early and reliable detection.

**Case Summary:**

A 4-month-old patient with familial hemophagocytic lymphohistiocytosis received an allogeneic hematopoietic cell transplant (HCT). Weekly CMV monitoring before and after HCT was performed using an in-house quantitative CMV PCR assay that targets the CMV *UL54* gene. Review of the amplification curves of PCR runs raised concerns about potential false-negative results. Sequencing of the patient sample identified nucleotide mutations within the probe-binding site, confirming the cause of the assay failure. These false-negative results led to delayed detection of CMV DNAemia before HCT and delayed initiation of CMV preemptive antiviral therapy after transplant.

**Conclusion:**

This case underscores the critical importance of rigorous and routine evaluation of CMV NAATs by clinical laboratories. Laboratories should recognize the limitations of NAATs and implement strategies to address them. In situations where laboratory findings conflict with clinical data, clinicians should critically assess a negative NAAT result. If CMV infection remains a concern, testing with an alternate PCR assay targeting a different gene should be considered.

## INTRODUCTION

Cytomegalovirus (CMV), a beta-herpes virus, causes significant morbidity and mortality in immunocompromised patients and is the leading cause of congenital infection and hearing loss in children in industrialized countries (1). Nucleic acid amplification tests (NAATs) are preferred for diagnosis and monitoring response to CMV-directed therapy. Primers and/or probes typically target highly conserved regions of the genome, such as glycoprotein B (*UL55*), DNA polymerase (*UL54*), matrix phosphoprotein (*UL83*), or major immediate-early (MIE) gene (2–4). However, nucleotide variability within *UL55* and MIE genes causing false-negative results has been reported (2, 5–7).

The Clinical Microbiology Laboratories at Nationwide Children’s Hospital has offered a laboratory-developed quantitative real-time PCR assay targeting a 61 bp fragment of the *UL54* gene from plasma as the standard-of-care molecular assay since 2003 (SOC-PCR) (3). Here, we describe a pediatric hematopoietic cell transplant (HCT) patient in whom this assay produced false negatives due to mutations in *UL54*, delaying initiation of CMV therapy.

## CASE PRESENTATION

A 4-month-old patient with familial hemophagocytic lymphohistiocytosis (homozygous *PRF1* mutation) received myeloablative conditioning and a 10/10 matched unrelated allogeneic HCT from a CMV-seropositive donor (recipient was CMV seropositive). Pre-HCT CMV NAAT testing was negative. The infant was considered at high risk of CMV reactivation, and per institutional protocol, preemptive prophylaxis management with twice-weekly CMV monitoring by SOC-PCR on plasma was performed post-transplant and was not detected.

**Fig 1:**
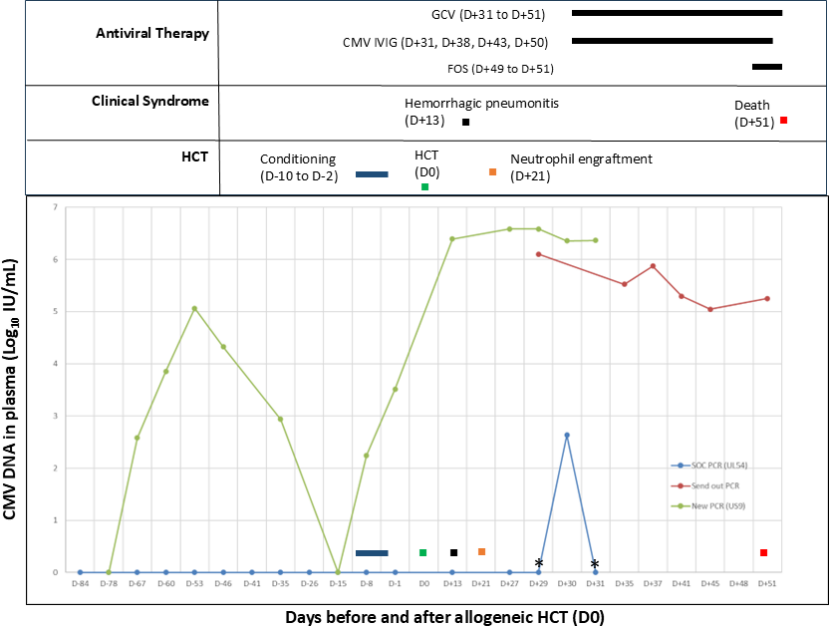
Plasma CMV viral loads by assay type and patient’s clinical course. *Viral loads were reported as indeterminate. CMV, cytomegalovirus; FOS, foscarnet; GCV, ganciclovir; HCT, hematopoietic cell transplantation; IVIG, intravenous immunoglobulin.

Thirteen days post-HCT (D + 13), the patient developed respiratory failure requiring intubation and progressive bilateral multifocal ground-glass opacities on imaging, consistent with alveolar hemorrhage and potential CMV pneumonitis. Infectious disease evaluation was negative, including blood cultures and adenovirus, herpes simplex virus, human herpes virus 6 PCRs and CMV-SOC PCR, respiratory tract secretions for bacterial cultures, adenovirus, HSV, *Pneumocystis jirovecii* PCRs and CMV-SOC PCR and multiplex respiratory panel (BioFire Respiratory Panel 2.0), as well as *Aspergillus* galactomannan (Platelia *Aspergillus* Ag). The evaluation was further expanded to include *Legionella* PCR, *Aspergillus* and *Mucorales* PCR on lower respiratory tract secretions with reflex to universal broad-range fungal PCR and *Legionella* urine antigen; all were negative. The patient achieved neutrophil engraftment on D + 21, and weekly CMV SOC-PCR monitoring remained negative (Fig. 1).

**Fig 2:**
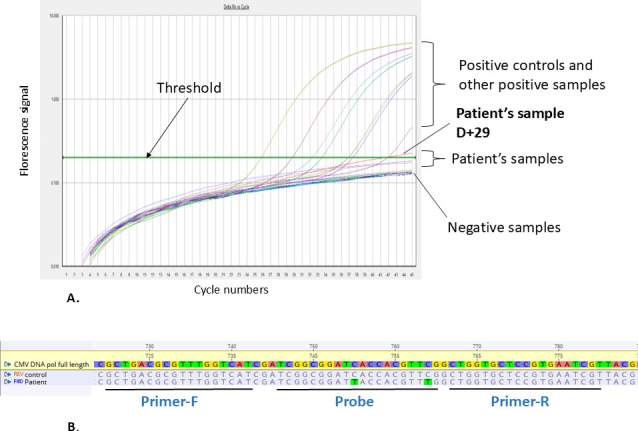
(**A**) CMV SOC-PCR amplification curves from the patient’s samples, assay controls, and other positive samples. Assay controls and other positive samples display typical sigmoidal shape with flat baseline, exponential rise, and plateau phase, while the patient’s samples displayed unusual amplification curves. (**B**) Alignment of the partial *UL54* gene: amplification region for the CMV SOC-PCR, from reference strain (NC_006273), assay control, and the patient’s sample. The location of the primers and probe is illustrated. The mutations within the probe-binding site found in the patient’s sample are highlighted in green.

On D + 29, the patient’s plasma exhibited an unusual amplification curve on the CMV SOC-PCR. The signal did cross the detection threshold and increased in amplitude slightly; it did not display logarithmic growth. Review of prior runs, although not crossing the threshold, showed similar atypical curves in samples previously reported as negative (Fig. 2A). These findings raised concern for false-negative results; therefore, the D + 29 sample was reported as indeterminate. The treating team was notified of this unusual result, and D + 29 plasma was sent to a reference laboratory using a PCR targeting the CMV *US9* gene. On the following day (D + 30), the plasma again demonstrated the same unusual amplification curve and was reported as positive after review. On D + 31, the reference laboratory reported a viral load of 1,260,000 IU/mL (6.1 log_10_ IU/mL) for the D + 29 sample, confirming our suspicion. Ganciclovir (5 mg/kg/dose every 12 hours) was started for CMV DNAemia (D + 31), and intravenous CMV immunoglobulin was given for possible CMV pneumonitis. Foscarnet (60 mg/kg/dose every 8 hours) was added to the treatment regimen. Weekly CMV monitoring was subsequently performed at the reference laboratory. Despite treatment, downtrending CMV PCR, and no evidence of hepatitis or retinitis, the patient remained critically ill. Plasma collected on D + 37 and D + 51 was sent to a reference laboratory to detect mutations within *UL97* and *UL54* genes by Sanger sequencing, and no mutations associated with ganciclovir or foscarnet resistance were found. On D + 51, the patient acutely developed refractory hypoxemia and asystolic cardiac arrest not responsive to resuscitation efforts.

The patient was CMV seropositive prior to the transplant; however, this was considered likely reflective of passive maternal antibody, and all CMV NAAT results pre-HCT were negative. To investigate further, we developed another CMV PCR targeting the *US9* gene (US9-PCR) testing all available remnant specimens (Fig. 1). We detected CMV DNAemia pretransplant and rising DNAemia post-HCT, demonstrating that the SOC-PCR had repeatedly produced false-negative results. This led to delayed detection of CMV DNAemia and delayed initiation of CMV preemptive therapy by 16 days.

One of the patient’s samples was sequenced in the region targeted by SOC-PCR, which revealed two C-T substitutions within the probe-binding site (Fig. 2B), confirming the false-negative results were due to sequence variation. Searches of publicly available databases (NCBI, accessed 8 June 2025) revealed three reports of these mutations: Belgium (GenBank KP745705, submitted 2015), Germany (GenBank JX512203, 2016), and Czech Republic (KY490065, 2017).

## DISCUSSION

HCT recipients at risk of CMV disease (all CMV-seropositive recipients and CMV-seronegative recipients with a CMV-seropositive donor) are monitored closely for CMV. The preemptive therapy strategy, involving monitoring for CMV DNAemia during at-risk periods and initiating antivirals when the CMV load reaches a certain threshold, has been proven highly effective in preventing CMV end-organ disease (8, 9). Furthermore, pretransplant CMV DNAemia is a strong risk factor for post-HCT CMV reactivation and associated complications (10, 11). Antiviral therapy to clear CMV DNAemia pre-HCT reduces the risk of reactivation post-HCT (10). With the US9-PCR, we detected CMV DNAemia in pretransplant samples and rising in DNAemia post-HCT, demonstrating that the SOC-PCR had repeatedly produced false negatives.

NAATs are inherently susceptible to false-negative results due to sequence diversity in the target genome. Extensive variability in the CMV genome and demonstration of multiple variants coexisting in an individual make assay design challenging (12, 13). In one prior report, it was found that samples from a leukemia patient undergoing antiviral therapy tested falsely negative on the COBAS assay (*UL54* target) for a period of 2 months. Retrospective testing with a dual target PCR assay (*UL54* and *UL55*) revealed high viral loads. These false-negative results delayed appropriate antiviral treatment. Although sequencing was not performed, the authors suggested that mutations within the primer/probe-binding regions were the probable cause (7). Additionally, a mismatch in the primer/probe region can lead to falsely low viral load. In the aforementioned study, viral loads for other samples tested were considerably lower for the UL55 assay than for the UL54 assay. Subsequent sequencing revealed a mismatch within the primer/probe for the UL55 assay (7).

Given the serious consequences of a false-negative CMV results, we implemented a surveillance program to screen SOC-PCR negative samples with the US9-PCR assay. Among >3,000 samples testing negative on the SOC-PCR that have been screened to date, four additional samples from four unique patients tested positive by US9-PCR assay. We sequenced one of these samples, revealing the same mutations as described in this case. This suggests that the primer/probe used in the SOC-PCR remains broadly inclusive and that strains carrying mutations compromising detection are relatively rare. Nevertheless, it is important for clinical laboratories and assay manufacturers to evaluate the inclusivity of their assays periodically. A 2009 study evaluating published CMV primers/probes identified a primer/probe set targeting *UL54*, which is the one used in our SOC-PCR, as one of the three most sensitive sets for CMV detection. *In silico* analysis revealed only one mismatch within the Towne strain (14). Our recent analysis of 462 published CMV sequences (NCBI, accessed 8 June 2025) identified 47 with at least one mismatch in the SOC-PCR binding region, although most involved a single mismatch unlikely to affect assay performance. This highlights the importance of ongoing evaluation of NAATs against newly submitted sequences. As mandated by the College of American Pathologists, clinical laboratories are required to evaluate laboratory-developed NAATs for compatibility with currently circulating microbial strains, thus ensuring continued diagnostic accuracy.

To enhance inclusivity, some CMV NAATs incorporate two distinct genomic targets as the likelihood of concurrent mutations in two conserved regions is low. Among eight FDA-approved CMV NAATs (Table 1), two utilize both *UL34* and *UL80.5* as targets. In one multicenter evaluation of the Alinity mCMV assay, only 8 of 336 positives were missed, and one discordant sample showed a higher load by Alinity than by a single-target reference PCR, suggesting target-region mismatch in the reference assay (15). Another study also demonstrated high agreement between the Alinity mCMV and a reference method (16). Single-target commercial assays have also shown high sensitivity and specificity (17–24) (Table 1), though rare false negatives do occur. A study of ARTUS CMV PCR demonstrated false lowering of viral loads with mismatches in the MIE target implicated as the cause (25). Notably, three FDA-approved assays use *UL54* alone, but their proprietary primer/probe sequences preclude *in silico* inclusivity checks by users.

**TABLE 1:** FDA cleared NAATs for detection and/or quantification of CMV^*a*^

Tests	Manufacturer	Technologies	Specimen type	Target(s)	Selected references
Abbott RealTime CMV	Abbott Molecular	Quantitative PCR	Plasma	UL34 and UL80.5 genes	(26–28)
Alinity M CMV	Abbott Molecular	Quantitative PCR	Plasma and whole blood	UL34 and UL80.5 genes	(15, 19)
Aptima CMV Quant Assay	Hologic, Inc	Quantitative TMA	Plasma	UL56 gene	(23, 24)
ARTUS CMV RGQ MDX Kit	Qiagen	Quantitative PCR	Plasma	Major immediate early gene (MIE)	(25, 29)
COBAS AmpliPrep/COBAS TaqMan CMV Test	Roche Molecular	Quantitative PCR	Plasma	UL54	(19–21)
COBAS CMV test	Roche Molecular	Quantitative PCR	Plasma	UL54	(19–21)
Simplexa Congenital CMV Direct	DiaSorin	Qualitative PCR	Saliva and urine	UL54	(18)
Alethia CMV DNA Amplification Assay	Meridian Bioscience	Qualitative LAMP	Saliva	UL33 gene	(22)

^*a*^ LAMP, loop-mediated amplification; TMA, template-mediated amplification.

Although not clinically available at the time, metagenomic next-generation sequencing (mNGS) could have allowed for broad pathogen detection in blood and pulmonary fluid samples in this case (30) as mNGS is not susceptible to sequence diversity and can detect mutated pathogens as efficiently as the wild-type strains.

To the best of our knowledge, CMV *UL54* mutations causing diagnostic failure have not been previously reported. Our case underscores the importance of rigorous and routine evaluation of CMV NAATs, even with well-characterized primer/probe sets. Laboratories should be aware of the limitations of NAATs and implement mitigation strategies, such as reviewing amplification curves for unusual patterns, periodic *in silico* analysis, thorough literature review, close communication with clinicians to identify potential false-negative cases, and considering use of multitarget assays. When laboratory findings conflict with clinical data, clinicians should critically assess a negative NAAT result and consider testing using an alternative gene target or mNGS for broader pathogen coverage.

## References

[1] Leber AL. 2024. Maternal and congenital human cytomegalovirus infection: laboratory testing for detection and diagnosis. J Clin Microbiol 62:e0031323. doi:10.1128/jcm.00313-23 38391188 PMC11005381

[2] Novak Z, Chowdhury N, Ross SA, Pati SK, Fowler K, Boppana SB. 2011. Diagnostic consequences of cytomegalovirus glycoprotein B polymorphisms. J Clin Microbiol 49:3033–3035. doi:10.1128/JCM.01039-11 21653769 PMC3147759

[3] Sanchez JL, Storch GA. 2002. Multiplex, quantitative, real-time PCR assay for cytomegalovirus and human DNA. J Clin Microbiol 40:2381–2386. doi:10.1128/JCM.40.7.2381-2386.2002 12089251 PMC120584

[4] Boppana SB, Ross SA, Novak Z, Shimamura M, Tolan RW Jr, Palmer AL, Ahmed A, Michaels MG, Sánchez PJ, Bernstein DI, Britt WJ, Fowler KB, National Institute on Deafness and Other Communication Disorders CMV and Hearing Multicenter Screening (CHIMES) Study. 2010. Dried blood spot real-time polymerase chain reaction assays to screen newborns for congenital cytomegalovirus infection. JAMA 303:1375–1382. doi:10.1001/jama.2010.423 20388893 PMC2997517

[5] Lengerova M, Racil Z, Volfova P, Lochmanova J, Berkovcova J, Dvorakova D, Vorlicek J, Mayer J. 2007. Real-time PCR diagnostics failure caused by nucleotide variability within exon 4 of the human cytomegalovirus major immediate-early gene. J Clin Microbiol 45:1042–1044. doi:10.1128/JCM.01109-06 17229861 PMC1829136

[6] Nye MB, Leman AR, Meyer ME, Menegus MA, Rothberg PG. 2005. Sequence diversity in the glycoprotein B gene complicates real-time PCR assays for detection and quantification of cytomegalovirus. J Clin Microbiol 43:4968–4971. doi:10.1128/JCM.43.10.4968-4971.2005 16207949 PMC1248473

[7] Herrmann B, Larsson VC, Rubin C-J, Sund F, Eriksson B-M, Arvidson J, Yun Z, Bondeson K, Blomberg J. 2004. Comparison of a duplex quantitative real-time PCR assay and the COBAS Amplicor CMV Monitor test for detection of cytomegalovirus. J Clin Microbiol 42:1909–1914. doi:10.1128/JCM.42.5.1909-1914.2004 15131148 PMC404600

[8] Boeckh M, Ljungman P. 2009. How we treat cytomegalovirus in hematopoietic cell transplant recipients. Blood 113:5711–5719. doi:10.1182/blood-2008-10-143560 19299333 PMC2700312

[9] Gutierrez V, Stanek J, Ardura MI, Song E. 2024. Cytomegalovirus viral load at initiation of pre-emptive antiviral therapy impacts cytomegalovirus dynamics in pediatric allogeneic hematopoietic cell transplantation recipients. Transpl Infect Dis 26:e14358. doi:10.1111/tid.14358 39185743 PMC11666870

[10] Zamora D, Xie H, Sadowska-Klasa A, Kampouri E, Biernacki MA, Ueda Oshima M, Duke E, Green ML, Kimball LE, Holmberg L, Waghmare A, Greninger AL, Jerome KR, Hill GR, Hill JA, Leisenring WM, Boeckh MJ. 2024. CMV reactivation during pretransplantation evaluation: a novel risk factor for posttransplantation CMV reactivation. Blood Adv 8:4568–4580. doi:10.1182/bloodadvances.2023012234 38924728 PMC11399585

[11] Fries BC, Riddell SR, Kim HW, Corey L, Dahlgren C, Woolfrey A, Boeckh M. 2005. Cytomegalovirus disease before hematopoietic cell transplantation as a risk for complications after transplantation. Biol Blood Marrow Transplant 11:136–148. doi:10.1016/j.bbmt.2004.11.016 15682075

[12] Ross SA, Novak Z, Pati S, Patro RK, Blumenthal J, Danthuluri VR, Ahmed A, Michaels MG, Sánchez PJ, Bernstein DI, Tolan RW, Palmer AL, Britt WJ, Fowler KB, Boppana SB. 2011. Mixed infection and strain diversity in congenital cytomegalovirus infection. J Infect Dis 204:1003–1007. doi:10.1093/infdis/jir457 21881114 PMC3164425

[13] Schnepf N, Dhédin N, Mercier-Delarue S, Andreoli A, Mamez A-C, Ferry C, Deback C, Ribaud P, Robin M, Socié G, Simon F, Mazeron M-C. 2013. Dynamics of cytomegalovirus populations harbouring mutations in genes UL54 and UL97 in a haematopoietic stem cell transplant recipient. J Clin Virol 58:733–736. doi:10.1016/j.jcv.2013.10.007 24183928

[14] Habbal W, Monem F, Gärtner BC. 2009. Comparative evaluation of published cytomegalovirus primers for rapid real-time PCR: which are the most sensitive? J Med Microbiol 58:878–883. doi:10.1099/jmm.0.010587-0 19502375

[15] Lee M, Albert E, Wessels E, Kim S-K, Chung H-S, Giménez E, Vreeswijk T, Claas ECJ, Tai YC, Reinhardt B, Sasaki MM, Navarro D. 2023. Multicenter performance evaluation of the Alinity m CMV assay for quantifying cytomegalovirus DNA in plasma samples. J Clin Microbiol 61:e0041523. doi:10.1128/jcm.00415-23 37728341 PMC10654106

[16] Kostera J, Hubbard M, Jackson D, Liesman RM. 2024. Evaluation of Alinity m CMV assay performance for detecting CMV in plasma, cerebrospinal fluid, and bronchoalveolar lavage specimens. Diagn Microbiol Infect Dis 109:116301. doi:10.1016/j.diagmicrobio.2024.116301 38723453

[17] Schneider M, Kollender K, Hilfrich B, Weiss R, Iftner T, Heim A, Ganzenmueller T. 2024. Evaluation of an automated real-time transcription-mediated amplification (TMA) assay for detection and quantification of cytomegalovirus DNA in different clinical specimens. J Clin Virol 171:105637. doi:10.1016/j.jcv.2023.105637 38218116

[18] Fernholz EC, Vidal-Folch N, Hasadsri L. 2023. Rapid and direct detection of congenital cytomegalovirus using a commercial real-time PCR assay. J Clin Microbiol 61:e0178122. doi:10.1128/jcm.01781-22 36786642 PMC10035318

[19] D’Costa J, Chibo D, Soloczynskyj K, Batty M, Sameer R, Lee E, Tran T, Mavroulis D, Gooey M, Williams E, Jackson K. 2025. Evaluation and comparison of three high throughput assays (Alinity m CMV, Aptima CMV Quant and cobas CMV) for quantifying CMV DNA in plasma samples. J Virol Methods 332:115068. doi:10.1016/j.jviromet.2024.115068 39551443

[20] Chiereghin A, Pavia C, Gabrielli L, Piccirilli G, Squarzoni D, Turello G, Gibertoni D, Simonazzi G, Capretti MG, Lanari M, Lazzarotto T. 2017. Clinical evaluation of the new Roche platform of serological and molecular cytomegalovirus-specific assays in the diagnosis and prognosis of congenital cytomegalovirus infection. J Virol Methods 248:250–254. doi:10.1016/j.jviromet.2017.08.004 28801056

[21] Pritt BS, Germer JJ, Gomez-Urena E, Bishop CJ, Mandrekar JN, Irish CL, Yao JDC. 2013. Conversion to the COBAS AmpliPrep/COBAS TaqMan CMV Test for management of CMV disease in transplant recipients. Diagn Microbiol Infect Dis 75:440–442. doi:10.1016/j.diagmicrobio.2013.01.014 23428458

[22] Gantt S, Goldfarb DM, Park A, Rawlinson W, Boppana SB, Lazzarotto T, Mertz LM. 2020. Performance of the alethia CMV assay for detection of cytomegalovirus by use of neonatal saliva swabs. J Clin Microbiol 58:e01951-19. doi:10.1128/JCM.01951-19 31969426 PMC7098765

[23] Sam SS, Rogers R, Ingersoll J, Kraft CS, Caliendo AM. 2023. Evaluation of performance characteristics of the aptima CMV quant assay for the detection and quantitation of CMV DNA in plasma samples. J Clin Microbiol 61:e0169922. doi:10.1128/jcm.01699-22 36719219 PMC9945493

[24] Lima A, Healer V, Rowe L, Silbert S. 2023. Performance evaluation of the Aptima CMV quant assay using plasma and non-plasma samples. J Clin Virol 164:105467. doi:10.1016/j.jcv.2023.105467 37126896

[25] Waggoner J, Ho DY, Libiran P, Pinsky BA. 2012. Clinical significance of low cytomegalovirus DNA levels in human plasma. J Clin Microbiol 50:2378–2383. doi:10.1128/JCM.06800-11 22518866 PMC3405616

[26] Schnepf N, Scieux C, Resche-Riggon M, Feghoul L, Xhaard A, Gallien S, Molina J-M, Socié G, Viglietti D, Simon F, Mazeron M-C, Legoff J. 2013. Fully automated quantification of cytomegalovirus (CMV) in whole blood with the new sensitive Abbott RealTime CMV assay in the era of the CMV international standard. J Clin Microbiol 51:2096–2102. doi:10.1128/JCM.00067-13 23616450 PMC3697667

[27] Tremblay M-A, Rodrigue M-A, Deschênes L, Boivin G, Longtin J. 2015. Cytomegalovirus quantification in plasma with Abbott RealTime CMV and Roche Cobas Amplicor CMV assays. J Virol Methods 225:1–3. doi:10.1016/j.jviromet.2015.08.010 26341060

[28] Furione M, Rognoni V, Cabano E, Baldanti F. 2012. Kinetics of human cytomegalovirus (HCMV) DNAemia in transplanted patients expressed in international units as determined with the Abbott RealTime CMV assay and an in-house assay. J Clin Virol 55:317–322. doi:10.1016/j.jcv.2012.08.017 22989927

[29] Michelin BDA, Hadzisejdic I, Bozic M, Grahovac M, Hess M, Grahovac B, Marth E, Kessler HH. 2008. Detection of cytomegalovirus (CMV) DNA in EDTA whole-blood samples: evaluation of the quantitative artus CMV LightCycler PCR kit in conjunction with automated sample preparation. J Clin Microbiol 46:1241–1245. doi:10.1128/JCM.01403-07 18272703 PMC2292973

[30] Zheng X, Zou W, Zou S, Ye J, Bao Z, Song Y. 2025. Diagnostic significance of metagenomic next-generation sequencing in immunocompromised patients with suspected pulmonary infection. Immunology 175:112–122. doi:10.1111/imm.13911 39988326 PMC11982602

